# Movement-related tactile gating in blindness

**DOI:** 10.1038/s41598-023-43526-8

**Published:** 2023-10-02

**Authors:** Maria Casado-Palacios, Alessia Tonelli, Claudio Campus, Monica Gori

**Affiliations:** 1 DIBRIS, University of Genoa, Genoa, Italy; 2 UVIP- Unit for Visually Impaired People, Italian Institute of Technology, Genoa, Italy

**Keywords:** Sensory processing, Human behaviour

## Abstract

When we perform an action, self-elicited movement induces suppression of somatosensory information to the cortex, requiring a correct motor-sensory and inter-sensory (i.e. cutaneous senses, kinesthesia, and proprioception) integration processes to be successful. However, recent works show that blindness might impact some of these elements. The current study investigates the effect of movement on tactile perception and the role of vision in this process. We measured the velocity discrimination threshold in 18 sighted and 18 blind individuals by having them perceive a sequence of two movements and discriminate the faster one in passive and active touch conditions. Participants’ Just Noticeable Difference (JND) was measured to quantify their precision. Results showed a generally worse performance during the active touch condition compared to the passive. In particular, this difference was significant in the blind group, regardless of the blindness duration, but not in the sighted one. These findings suggest that the absence of visual calibration impacts motor-sensory and inter-sensory integration required during movement, diminishing the reliability of tactile signals in blind individuals. Our work spotlights the need for intervention in this population and should be considered in the sensory substitution/reinforcement device design.

## Introduction

Since birth, humans interact with their environment through actions. For this to occur, sensory and motor information integration is required^1^. For example, the motor and sensory functions are intimately interrelated at the level of the hands, since the primary somatosensory cortex (S1) is one of the input sources to the motor cortex^2^. Active touch is defined as voluntary movement elicited by the participant and relies on motor-sensory interaction and inter-sensory processes, as it involves cutaneous senses, kinesthesia, and proprioception^3–5^. On the contrary, passive touch involves only inter-sensory processes^1,6^.

Some authors report better tactile perception of a stimulus when there is movement between it and the skin, compared to when it is statically presented^7,8^. Surprisingly, when the movement of our own body creates this movement, some electrophysiology studies show that the somatosensory cortex experiences a suppression of afferent information^6,9,10^. This phenomenon—called tactile suppression or movement-related tactile gating—might help limit the amount of sensory information processed by the cortex^6,9^. It has been hypothesised that this process is part of the central nervous system’s (CNS) functional sensory modulations and occurs at different levels of the somatosensory pathway^6,11,12^. Whether tactile gating leads to a general reduction in tactile perception or stems from a sensory prediction, as happens with self-generated touches^13–16^, is a topic under debate^11^, with some authors suggesting that tactile gating is an independent process unrelated to any sensory anticipation^17^. However, there is strong evidence that tactile gating is influenced by a forward model^11^.

This model states that when a movement is generated, along with the descendent motor command, we produce an internal representation of that command called “efference copy”. In other words, we produce an internal prediction of the sensory consequences resulting from our actions. Then, this efference copy is compared with the feedback conveyed by the sensory receptors or “reafference”. Sensory gating occurs if the prediction and the actual perception are coherent. The difference is interpreted as afferent information from the outside world, called “exafference”, which is the information considered relevant and thus transmitted to the following processing levels^18,19^. For example, when tracing the outline of an object with your fingertip, the brain may selectively attend to the tactile signals that provide shape or texture information while suppressing irrelevant background tactile inputs.

A precise exafference requires a precise efference copy and a correct integration between the feedback perceived by our cutaneous receptors, muscles, and joints. In other words, touch, proprioception, kinesthesia, and motor information must be integrated to efficiently estimate which information is self-generated from the one generated externally. Interestingly, some of these cognitive processes might be vulnerable to lacking one sensory modality. For instance, some authors found that the lack of vision is responsible for increased muscle sense reliability when guiding goal-directed movement^20^. Moreover, it has been suggested that proprioceptive spatial representation is delayed or dramatically weakened without visual calibration over the haptic and auditory modalities early in life^21,22^. The integration between touch and proprioception also seems to be affected by the loss of sight. Research in blind individuals^23^ shows, using a somatic rubber hand illusion paradigm (i.e., illusion experienced by sighted participants in which the hand ownership is shifted from the real hand to the rubber hand^24^), that blind participants do not experience this illusion. The authors explained their results based on differences in the integration of multisensory information at the central level. They suggest that blind individuals lack the ability to re-map tactile feedback into an external frame of reference, a skill commonly observed in sighted individuals. As a result, blind individuals are believed to be unable to integrate the simultaneous touch of a rubber hand and their hand, which is the mechanism that enables sighted individuals to experience the illusion^23,25^. This reduced interaction between multisensory cues has been extensively reported also between touch and hearing. To illustrate this, Hötting and Röder (2004) observed that congenitally blind participants, when compared to sighted individuals, were less influenced by task-irrelevant stimuli in an auditory-tactile illusion task^26^. In this line, Occelli et al. (2011) had the same results in a ventriloquist effect experiment, in which spatially discrepant information from one sensory modality biases the localization of another stimulus perceived simultaneously from another modality. This study found a reduced effect in the congenitally blind group, concluding the presence of a lessened audio-tactile interaction in the absence of visual input early in life^27^. A possible explanation can be found in the cross-modal calibration theory. According to this model, before the consolidation of multisensory processing abilities, which implies decoding across distinct reference frames and identifying cues that can be merged into a unified percept^28^, it is imperative a continuous process of recalibration across different perceptual systems during the developmental stages. This recalibration process is driven by cross-sensory comparisons, wherein the most informative sense guides the calibration of others^29^. This dynamic process leads to optimal integration, helping to create a multisensory representation of the world. For example, in the case of spatial perception, vision is the most reliable sense^30^, and due to that, the missing visual input could lead to an altered multisensory interaction^26,31–33^, which has been related to spatial developmental impairments or delays^34^. This makes us wonder if, in a spatial task, vision might affect the motor-sensory and inter-sensory integration processes required during an action. Unexpectedly, despite all these findings and the important role that active touch plays in the daily life of a blind person, the effect that voluntary movements have on this population is a neglected topic.

With the present work, we aimed to study the effect of movement on perception and how the lack of visual input can modulate that effect. For this purpose, we used dynamic stimuli. As mentioned above, the presence of a movement between the stimulus and the skin has been associated with a more accurate tactile perception^6^. A dynamic stimulus allows us to have a movement between the stimulus and the hand even when it is in a fixed position, allowing us to dig into the role of active movement. Determining tactile performance during passive versus active movement would enable us to define how motor and sensory processes interact when vision is absent. To do this, we developed an experiment to measure the velocity discrimination threshold in blind and sighted participants in a two-alternative-force-choice (2AFC) task.

## Methods

### Participants

A power analysis was performed to decide the sample size using the mean and the standard deviation (SD) obtained from a study about event-time perception in tactile perception^35^ for the active and passive movement conditions. We used this paper because it found a significant difference between passive and active tactile perception with the same device used in our experiment. To calculate our sample size, we computed a power analysis via the function “pwr.t.test” (package “pwr”) in RStudio (Version 1.4.1106; 2021), which uses the Cohen d value extracted from the above paper to predict the sample size needed according to the desired alpha and power. The value of Cohen d calculated from Tomassini’s experiment was 1.48, and the sample size was 8. The power analysis returned a sample size of 11 participants, considering an alpha of 0.05 and a power of 0.9. To compensate the lack of homogeneity in terms of blindness duration, we decided to increase the number to 18 participants per group. Therefore, 18 blind individuals (10 women and 8 men), aged between 22 and 59 (mean age ± SD: 41.67 ± 11.9 years) and 18 age-matched sighted controls (12 women and 6 men; age mean ± SD: 35.11 ± 11.72) participated in our study (no age difference: t(34) = 1.67, *p* = 0.105). The range in the onset of blindness is from 0 to 46 years (11.11 ± 15.76 years). The clinical details of the blind individuals tested are summarised in Table 1. All subjects reported no history of cognitive or motor-sensory deficits (except blindness).

**Table 1:** Clinical details of the blind population tested, including the gender, age, pathology, blindness onset, visual perception, and frequency of braille reading.

Participant	Gender	Age	Pathology	Blindness onset	Visual perception	Frequency braille reading
S1	F	22	Retinopathy of prematurity	0	Lights and shadows	Rarely
S2	F	34	Retinopathy of prematurity	0	No	Often
S3	F	50	Retinopathy of prematurity	0	Lights and shadows	Always
S4	F	32	Hereditary retinal dystrophies Retinitis pigmentosa	0	Lights and shadows	Rarely
S5	F	32	Retinopathy of prematurity	0	No	Occasionally
S6	M	49	Congenital atrophy of the optic nerve	0	No	Occasionally
S7	M	52	Optic nerve subatrophy for a benign tumor	40	Lights and shadows	Never learnt
S8	M	29	Leber Amaurosis	0	No	Rarely
S9	F	56	Retinitis pigmentosa	35	Lights and shadows	Never learnt
S10	M	49	Congenital glaucoma	6	No	Often
S11	M	33	Corneal opacity	17	No	Often
S12	M	43	Leber Amaurosis Retinitis pigmentosa	26	Lights and shadows	Rarely
S13	F	43	Atrophy of the optic nerve	0	No	Rarely
S14	F	54	Retinitis pigmentosa	0	No	Rarely
S15	M	59	Glaucoma	0	No	Never
S16	F	56	Dystrophies primarily involving the retinal pigment epithelium Leber’s congenital amaurosis	46	No	Occasionally
S17	M	33	Degenerative oculopathy of both eyes	20	Light and shadows With strong contrast even colors, a field of view < 1%	Never
S18	F	24	Alstrom syndrome Retinitis pigmentosa	10	Lights and shadows	Rarely

The research protocol was approved by the local health service’s ethics committee (Comitato Etico, ASL3 Genovese, Italy) (Comitato Etico Regione Liguria, Genoa, Italy; Prot. IIT_UVIP_COMP_2019 N. 02/2020, 4 July 2020) in line with the Declaration of Helsinki. All participants signed informed consent.

### Design and procedure

**Figure 1:**
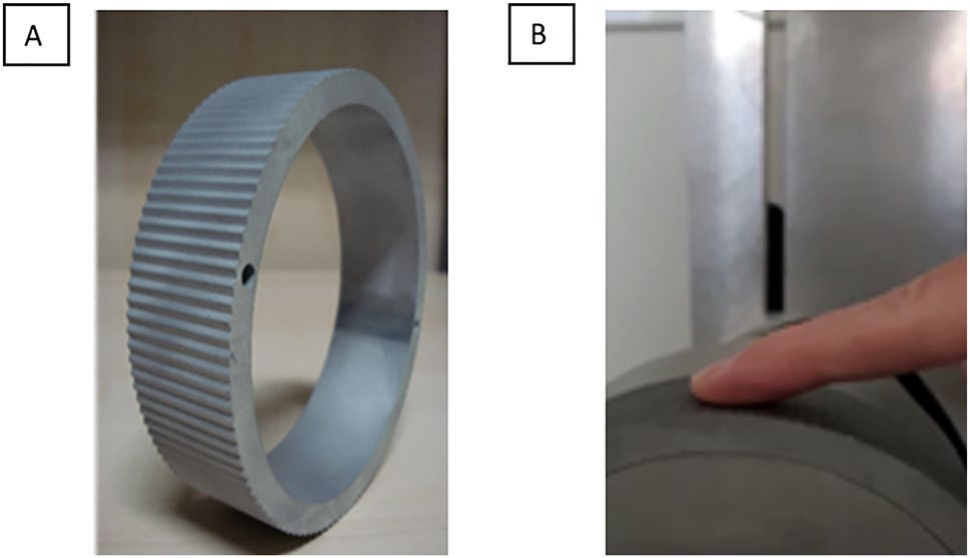
Experimental setup. The wheel’s movement provided the tactile stimulus (**A**), which presents a sinusoidal grating of 10 cycles/cm. (**B**) represents the position of the finger in the wheel.

A custom-made device (Fig. 1)^35–37^ produced the tactile stimuli, controlled by a custom Matlab code (R2020b, The MathWorks, USA). The wheel attached to the device had a 10 cycles/cm sinusoidal grating and could move at different velocities.

During the experiment, participants sat in a chair with their body’s midline aligned with the center of the wheel. The position of the finger and the orientation of the wheel is represented in Fig. 1. The tactile area stimulated was the index finger of the right hand, and both sighted and blind participants were blindfolded during the experiment, ensuring that the variable of sight was fully removed from both groups. Participants perceived two movements presented subsequently: a standard with a fixed velocity and a comparison whose velocity was random. The participants were tasked with discriminating which of the two stimuli was faster by reporting the answer verbally to the experimenter.

The first stimulus presented had a velocity of 3cm/s (standard) with a duration of 1 s. Then, there was a pause for 500 ms, followed by a second stimulus with a different speed that also lasted 1 s (comparison). The comparison speed was selected using an adaptive algorithm, QUEST^38^ to guarantee that all trials converged on the threshold, ensuring a well-sampled psychometric function for estimating our dependent variable.

Participants performed the task twice in two experimental conditions: (1) passive touch, in which participants kept their finger in a fixed position with the wheel moving beneath it, and (2) active touch, in which participants had to move their finger in the opposite direction of the wheel, while it was moving. The active movement was controlled through instructions^39^, asking the participants to maintain a trained velocity of approximately 3 cm/s, the same velocity as our standard speed. A sound-isolating headphone was provided to eliminate the noise produced by the device to ensure the processing of only the tactile information. Each participant performed 30 trials per condition.

### Data analysis

Cumulative Gaussians were used to fit the data for each condition, yielding the threshold and PSE estimates from the best-fitting function’s standard deviation and the mean. Standard errors for the threshold and the PSE estimates were calculated by bootstrapping^40^. We used the threshold or just noticeable difference (JND) as a dependent variable for this work. The JND represents the minimum velocity needed to accurately discriminate the faster stimulus in 75% of the trials.

Later, we looked at the data to detect possible outliers, which are defined as those whose JND values are greater than the third quartile and more than 1.5 times the interquartile range or lower than the first quartile minus 1.5 times the interquartile range using JASP 0.16.0.0. This criterion was defined a priori. Four outliers were detected with a worse performance (i.e. higher values of the JND): three from the blind group and one from the sighted control one. One blind participant was an outlier in both the active and passive conditions, another just in the active, and a third in the passive. The sighted was an outlier just in the passive condition. In addition to fulfilling the outlier criteria, the visual inspection of the data confirmed their poorer performance and suggested that those participants could not adequately execute the assigned task. Due to that, 32 participants (15 blind and 17 sighted) were included in the analysis.

Due to the variability in the blindness duration, to decide whether divide the blind sample, we computed a Pearson correlation analysis between the JND of each condition (Passive, Active) and the blindness duration using the “cor.test” in R (package “*stats*”).

After verifying the normality of our sample using the Jarque Bera test (see supplementary materials), we performed a Mixed two-way ANOVA considering the condition (Passive, Active) as within factor and group (Blind, Sighted) as between factor. The effect size was calculated by partial eta squared (h2P). We performed Post-hoc comparisons with paired t-tests for the within factor and unpaired t-tests for the between factor. In this case, Cohen’s d was calculated as the effect size using the “cohensD” function in R (package “lsr”). Probabilities were considered significant when lower than 0.05 after Bonferroni correction.

Additionally, given that the average age of the blind group in our sample is slightly higher (albeit not significantly) than that of the sighted group, we decided to investigate whether age could influence our results. Consequently, to ensure the validity of the results, we executed a linear model (LM) analysis to study a possible linear relationship between the participant’s age and performance using the lm function (Package “stats”). First, using the JND as a response variable, we computed the linear model for each condition (Passive, Active) separately, being the between-subject fixed effects of the model the group (Blind, Sighted), and the age of the participants. In a second step, we applied this model distinctly for each group (Blind, Sighted), including age as the only between-subject effect. An ANOVA was performed to estimate the significance of the model’s effects. To compute the confidence interval for the R^2^ value resulting from each model, we bootstrapped it using the boot function (Package “boot”). The R^2^ value reported in this paper comes from the bootstrap analysis.

The effects of braille reading in the results were controlled by implementing a LM including frequency of braille reading (“Always”, “Often”, “Occasionally”, “Rarely”, “Never” and “Never Learnt”), as the between-subject effect. As in the previous LMs, ANOVA was used to estimate the significance of the model’s effects. To compute the confidence interval for the R^2^ value resulting from each model, we bootstrapped it using the boot function (Package “boot”). The R^2^ value reported in this paper comes from the bootstrap analysis. The results of these analyses can be found in the supplementary materials.

### Ethics approval

The research protocol was approved by the local health service’s ethics committee (Comitato Etico, ASL3 Genovese, Italy) in line with the Declaration of Helsinki. All participants signed informed consent.

## Results

No correlations were found between the blindness duration and the JND value for the passive condition (t(13) = − 1.443, *p* = 0.173, r = − 0.371, 95% CI [− 0.742, 0.174]) or the active condition (t(13) = − 0.056, *p* = 0.956, r = − 0.015, [− 0.524, 0.501]), suggesting the performances in the blind group were due to a lack of vision regardless of the duration of their blindness. Given this, we decided to include all blind participants in one group.

**Figure 2:**
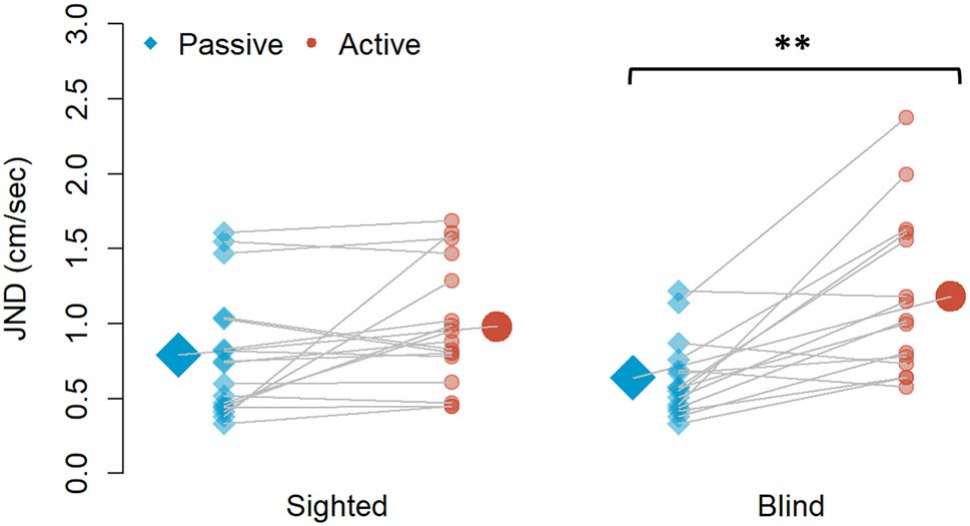
Just noticeable difference (JND) of sighted and blind participants for each condition: passive marked with blue diamonds and active with red circles. The JND in the active condition (red circle) was higher than in the passive one (blue diamond). However, considering the passive condition as a baseline, only blind individuals performed significantly worse when there was an active movement. *p, 0.05. **p, 0.01.

The Mixed ANOVA on the JND showed a significant main effect of condition (Passive, Active) (F(1,30) = 19.969, *p* < 0.001, η2P = 0.40, 95% CI [0.18, 1]) with a higher JND value during active touch, implying lower precision compared to the passive one for both groups. We also found a significant effect of the interaction between the group (Blind, Sighted) and the condition (Passive, Active) (F(1,30) = 4.879, *p* = 0.035, η2P = 0.14, [0.01, 1]). Specifically, we observed that the blind participant group showed a significant difference between their performance in the active and passive conditions (t(20.241) = − 3.467, p adjusted (adj) = 0.005, Cohen’s d = 1.266, [0.018, 2.449]), while this difference was not significant for the sighted group (t(31.981) = − 1.337, p adjusted (adj) = 0.381, Cohen’s d = 0.459, [− 0.533, 1.423]), meaning that only the blind group showed a significant decrease in precision in the active condition (Fig. 2). Moreover, when we compared the two groups, their precision did not significantly differ in the passive (t(27.303) = 1.236, p adjusted (adj) = 0.454, Cohen’s d = 0.426, [− 0.280, 1.125]), and neither in the active conditions (t(25.833) = − 1.163, p adjusted (adj) = 0.511, Cohen’s d = 0.419, [− 0.287, 1.118]), meaning the significant difference in the blind group is not due to a significantly better precision of this group during passive touch or a significant worse perception during active touch, but a significant performance deterioration due to the active movement.

**Figure 3:**
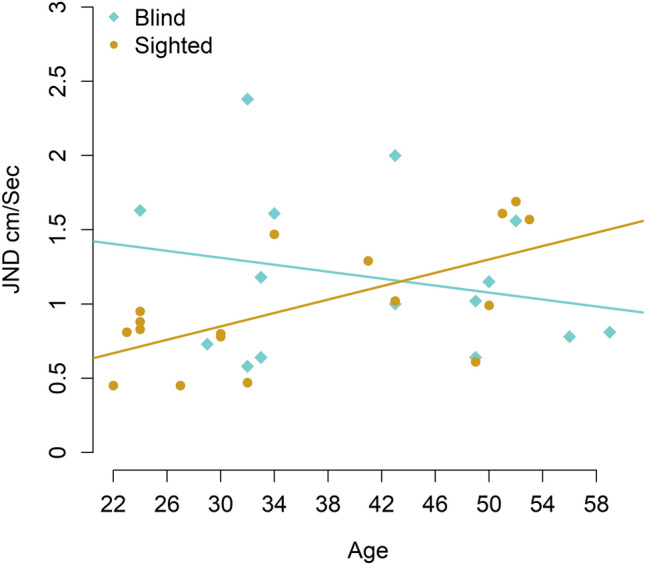
Linear model (LM) analysis between the participants’ age (blind and sighted) and their performance in the active condition. We applied an LM distinctly for each group (blind–blue diamonds, sighted–yellow circles), including the age as fixed effect. In this figure, the blue line represents the regression line of blind individuals, while the yellow one corresponds to the regression line of sighted participants. In the model for the blind group, changes in the JND were not significant. However, age in the sighted group has a significant main effect, showing an increased threshold in older participants.

In the first LM, we analysed the passive tactile performance of sighted and blind participants by considering the group and the age as fixed effects, with none being significant (R^2^ = 0.148, *p* = 0.206, CI 95% [0.062, 0.539]). We replicated this analysis for the active tactile condition in the second LM. A trend effect of the interaction group-by-age was revealed using this analysis (R^2^ = 0.231, *p* = 0.058, [0.099, 0.559]). To further investigate the impact of age on the JND obtained in the active condition, we applied two LMs separately for each group, one for the blind group and one for the sighted group (See Fig. 3). In both, we considered age as a fixed effect. In the model for the blind group, there was no significant effect (R^2^ = 0.055, *p* = 0.401, [0.0002, 0.378]). Interestingly, there is a significant main effect of age in the sighted group (R^2^ = 0.413, *p* = 0.005, [0.072, 0.786]), showing an increased threshold in older participants. These results suggest that precision in tactile perception during active movements in the blind group is not dependent on age, which is not the case for their sighted counterparts.

## Discussion

Perception and action are linked through motor-sensory and inter-sensory integration. Active touch is the expression of this process, essential for interacting with the environment^1^. Despite that, how this integration might be affected by the absence of vision is a neglected topic. Here we hypothesised a similar performance in active and passive touch due to compensatory mechanisms during self-generated movements^6^ in the sighted participants, while a worse precision in the active condition in blind individuals due to the absence of visual calibration^21^. However, our two main results were: (i) a generalised decrease in precision in the active condition compared to the passive one, and (ii) this precision reduction was enhanced in the blind group.

This study investigated the ability to discriminate velocity using tactile information and how the lack of visual input can modulate active touch. Using a physical wheel to provide dynamic tactile stimuli, we measured the precision in discriminating between different velocities in blind and sighted individuals in passive and active conditions. In the former case, the participant felt the wheel’s movement with their finger fixed; in the latter case, participants had to move the finger in the opposite direction of the wheel.

To explain the generalised lower precision in the active compared to the passive condition, we can refer to the so-called movement-related tactile gating. According to this phenomenon, body movements would reduce the amount of sensory information processed by the somatosensory cortex, leading to worse encoding^1,6,41^, with previous works reporting higher detection thresholds^42,43^ or worse detection rates during self-generated movements^44^. Despite this effect, a clear general superior precision of the passive touch in tactile perception compared to active touch is not reported. Some studies demonstrate that the use of motor strategy might enhance the performance during active touch, for example, by adapting the speed of the movement, the gating, and the optimal orientation of the digits during the exploration. Furthermore, the possible increased activation of central components, such as attention or motor set, might also contribute to this enhanced performance (for a review see^7^). These two factors might compensate wholly or partially for the gating effect, bearing a similar perceptual experience in most tasks. In our experiment, some of the motor strategies could not be used, as the speed of the movement and the finger’s orientation was fixed, not compensating for the reduced information processed by the somatosensory cortex. This could explain why, in our case, the precision was lower in the active condition for both groups.

The second result is related to different behaviors between blind participants and the control group. Specifically, only blind participants had significantly worse precision during the active touch condition than the passive one. The underlying mechanism of the gating of the sensory information observed during self-generated movements might be related to the production of the efference copy^45–48^. As previously mentioned, the efference copy is the internal representation of the descending motor command created during a self-generated movement, enabling us to predict the sensory feedback of the self-movement. This anticipated sensory feedback is compared with the real sensory feedback, and the difference is the relevant information from the outside environment that needs to be processed^11,12^. This process has a functional role, allowing us to discriminate between afferents related to the external world and those originating from our movements^19^. Because of that, a noisy efference copy mechanism can originate an imprecise anticipated sensory feedback, resulting in a compromised haptic performance^1^. It happens with children^1,41,49^, whose motor development and ability to integrate information do not reach adult-like levels until 8–12 years of age^1^. This highlights that reliable information and integration are mandatory requirements in this process.

On the one hand, in blind individuals, there is an inaccurate proprioceptive spatial representation^21^. On the other hand, the absence of visual calibration also induces reduced multisensory integration and interaction between senses^23,34,50,51^. Our results suggest that this alteration in multisensory processing might also be extended to the motor-sensory and inter-sensory integration, being responsible for the non-specific gating of somatosensory cortical activity during active movement. This affects the neural computations involved in the processing and comparison of active touch feedback leading to significantly worse performance in the active condition. This implies that the lack of visual input has even wider consequences than expected, suggesting its calibration may play a key role in our perception during active touch explorations, diminishing the tactile feedback reliability in blind individuals.

Moreover, we would like to highlight that the performance of the blind participants was not influenced by blindness duration. The distinction between early and late blind is a topic under debate: while some authors describe a similar performance between the late blind and the sighted individuals^26,27,50^, others do not find this similarity^51^, suggesting that visual input may also be a requirement for some cognitive processes’ maintenance. Recent works supported this hypothesis, implying that visual deprivation over the years gradually modulates neural responses associated with space representation^52,53^. Although our sample’s minimum period of blindness duration was just 10 years, our results suggest that the lack of visual calibration during this time might be enough to alter motor-sensory and inter-sensory integration.

As a final point, our study showed that precision in the tactile perception during active movements in blind individuals remains intact over the years, while sighted participants experience a decline with age. This is in line with Norman and colleagues' (2022) work, which reported age’s adverse effect on tactile precision in sighted individuals when judging speed^54^. Curiously, Legge and collaborators (2008) compared the performance of sighted and blind participants finding a high acuity retained through years during active exploration in blind participants while the one of their aging sighted peers decreased by nearly 1% per year. They discussed their results proposing that the use of active touch by individuals with visual impairments in daily activities fosters the preservation of tactile sensitivity throughout the lifespan^55^. These findings allow us to rule out a possible impact of age differences between the blind and the sighted group as the responsible of the poorer performance observed during active touch, compared to passive touch, in the former group. This suggests that is the altered tactile gating the responsible of the decreased precision during active movements in the blind participants.

One possible limitation of the present study is that, although the observed alterations in the JND align with the findings reported by Kilteni and Ehrssons (2022), who attributed these changes to movement-related tactile gating, it might be possible that with our paradigm, both sensory attenuation (i.e., touch generated by our own body) and gating (i.e., external touch perceived during movement) are triggered. Moreover, whether the inaccurate predictive feedback might prevent crucial sensory information from being processed by higher cortical levels due to a more robust tactile suppression or, on the contrary, might allow non-informative signals arising from the motor command trigger by a lower strength tactile attenuation, has yet to be explored. It is also important to mention that this sensory gating might be modulated and alleviated by the task's context or relevance. For instance, Juravle and colleagues (2013) found a weakened tactile suppression during tactile exploration when the hand’s movements were needed to gather the tactile information^56^. Some authors even suggest a possible movement-related sensory facilitation^57^. Due to this, since the movement elicited during our experimental task was irrelevant, further investigation of this topic in a more ecological context is needed.

## Conclusion

To conclude, our work shows that visual calibration over the haptic modality significantly impacts our perceptions during self-movement, as its absence might be responsible for an inaccurate anticipated sensory feedback, leading to an inexact gating of somatosensory information. Our data also suggest that this process is extremely sensitive to the lack of vision, as 10 years of visual deprivation seems to be enough for this alteration to occur. Based on these findings, blind individuals may experience compromised precision when faced with unfamiliar tasks requiring active explorations. In a world quickly developing and bringing new products into the market, this must be considered in future procedures to foster sensory substitution/reinforcement devices, like tactile gloves or haptic displays. Nonetheless, these findings are especially important in the rehabilitation field, stressing the urgency of intervention in this population.

### Supplementary Information

Supplementary Information.

## Data Availability

Raw data is available on Zenodo repository (https://doi.org/10.5281/zenodo.6581096). The code will be sent upon request by contacting Maria Casado-Palacios.
