# Supplementary material for: Movement-related tactile gating in blindness
Source: Sci Rep. 2023 Oct 2;13:16553. doi: 10.1038/s41598-023-43526-8 (PMC10545755; doi:10.1038/s41598-023-43526-8)
Supplement: Supplementary file 1 — Supplementary Information. [file 41598_2023_43526_MOESM1_ESM.pdf]

Movement-Related Tactile Gating in Blindness

Maria Casado Palacios[1,2], Alessia Tonelli [2], Claudio Campus[2] and Monica Gori[2]

[1] DIBRIS Department, Università di Genova, Genova 16145, Italy

[2] U-VIP, Unit for Visually Impaired People, Istituto Italiano di Tecnologia (IIT), Genova 16152, Italy

Normality assumptions

Regarding Just noticeable difference (JND) variable, Jarque Bera Test confirmed that normality assumption was respected for both groups in both conditions (Passive blind: Xsquared = 2.762, df = 2, p = 0.251; Active blind: X-squared = 1.701, df = 2, p = 0.427; Passive sighted: X-squared = 2.230, df = 2, p = 0.328; Active sighted: X-squared = 1.214, df = 2, pvalue = 0.545), thus we conducted a Mixed two-way ANOVA.

Effects Braille reading

To explore the possible effects of the frequency our blind participants use braille over their performance we applied a LM for each condition. No significant effect was found neither for passive ($R2=.153$, $p= .886$, [.067, .823]) or active touch condition ($R2=.096$, $p= .958$, [.048, .78]). These results suggest that precision does not depend on the frequency our participants use braille.
